# Pediatric intramedullary schwannoma with syringomyelia: a case report and literature review

**DOI:** 10.1186/s12887-018-1341-2

**Published:** 2018-11-28

**Authors:** Keda Wang, Jizong Zhao, Yan Zhang, Yibing Su

**Affiliations:** 1 Department of Neurosurgery, Beijing Jishuitan Hospital, NO31, Xinjiekou East Street, Xicheng District, Beijing, 100035 China; 2 Department of Neurosurgery, Beijing Tiantan Hospital, Capital Medical University, NO6, Tiantan Xili, Dongcheng District, Beijing, 100050 China; 3 China National Clinical Research Center for Neurological Diseases, NO6, Tiantan Xili, Dongcheng District, Beijing, 100050 China

**Keywords:** Intramedullary tumor, Schwannoma, Syringomyelia, Spinal deformity, Pediatrics

## Abstract

**Background:**

Intramedullary schwannomas without neurofibromatosis are exceedingly rare. They are rarer in children with only 8 cases reported so far. The association of intramedullary schwannomas with syringomyelia is also rare. Here, we present a case of intramedullary schwannoma with syringomyelia treated surgically in an 9-year-old boy.

**Case presentation:**

We reviewed the clinical course of a 9-year-old boy, who presented with both lower extremity weakness of 6-month duration. Neurophysical examination revealed a decreased sensation below the T10 dermatome. Magnetic resonance imaging (MRI) showed an well-demarcated intramedullary lesion located at the level of T8 vertebra with isointensity on T2WI and hypointensity on T1WI, which was homogeneous enhanced after gadolinium injection. There was associated syringomyelia extending from T7 down to the level of T10. A mild scoliotic deformity was also observed. The lesion was totally resected after an T7-T8 laminoplasty. Histopathological findings were consistent with schwannoma. Postoperative MRI did not reveal the presence of a residual tumor with syringomyelia reducted. By 2 weeks after treatment, the patient had experienced nearly complete recovery. Management with external bracing was performed on this patient for 3 months after surgery to prevent spinal deformity. However, mild spinal kyphosis occurred 5 months after surgery, and a progressive postoperative spinal kyphosis was observed during these 3 years of follow-up. Continued conservative management with observation was performed as there is no association with functional decline and impairment in health-related quality-of-life measures.

**Conclusion:**

Although extremely rare and uncommonly associated with syringomyelia, schwannomas need to be considered in the preoperative diagnosis of solitary intramedullary tumors in children as total resection can be achieved improving surgical outcome; Pediatric patients should be monitored closely for the development of spinal deformity following resection of intramedullary schwannoma, particularly possessing preoperative scoliotic deformity and/or tumor-associated syringomyelia.

## Background

Schwannomas are the most common primary tumors of the spine, and are usually located intradurally extramedullary [1]. Intramedullary schwannomas account for 0.3% of intraspinal tumors and 1.1% of intraspinal schwannomas [2, 3]. Because of the rarity of this tumor, only about 60 cases have been reported in the spinal cord without neurofibromatosis and generally present in the fourth decade of life [1, 4, 5]. Pediatric intramedullary schwannoma without neurofibromatosis is extremely rare with only eight cases reported so far [6–13]. Since the risk involved in removal and the surgical strategy are different for intramedullary schwannomas and intramedullary astrocytomas, awareness of this possible diagnosis will help establish the optimum medical and surgical treatment and the prognosis [14]. In addition, the association of intramedullary schwannomas with syringomyelia can be found but are uncommon [8]. Here, we present a surgically treated case of pediatric intramedullary schwannoma with syringomyelia, which developed a progressive spinal kyphosis during these 3 years of postoperative follow-up.

## Case presentation

**Fig. 1:**
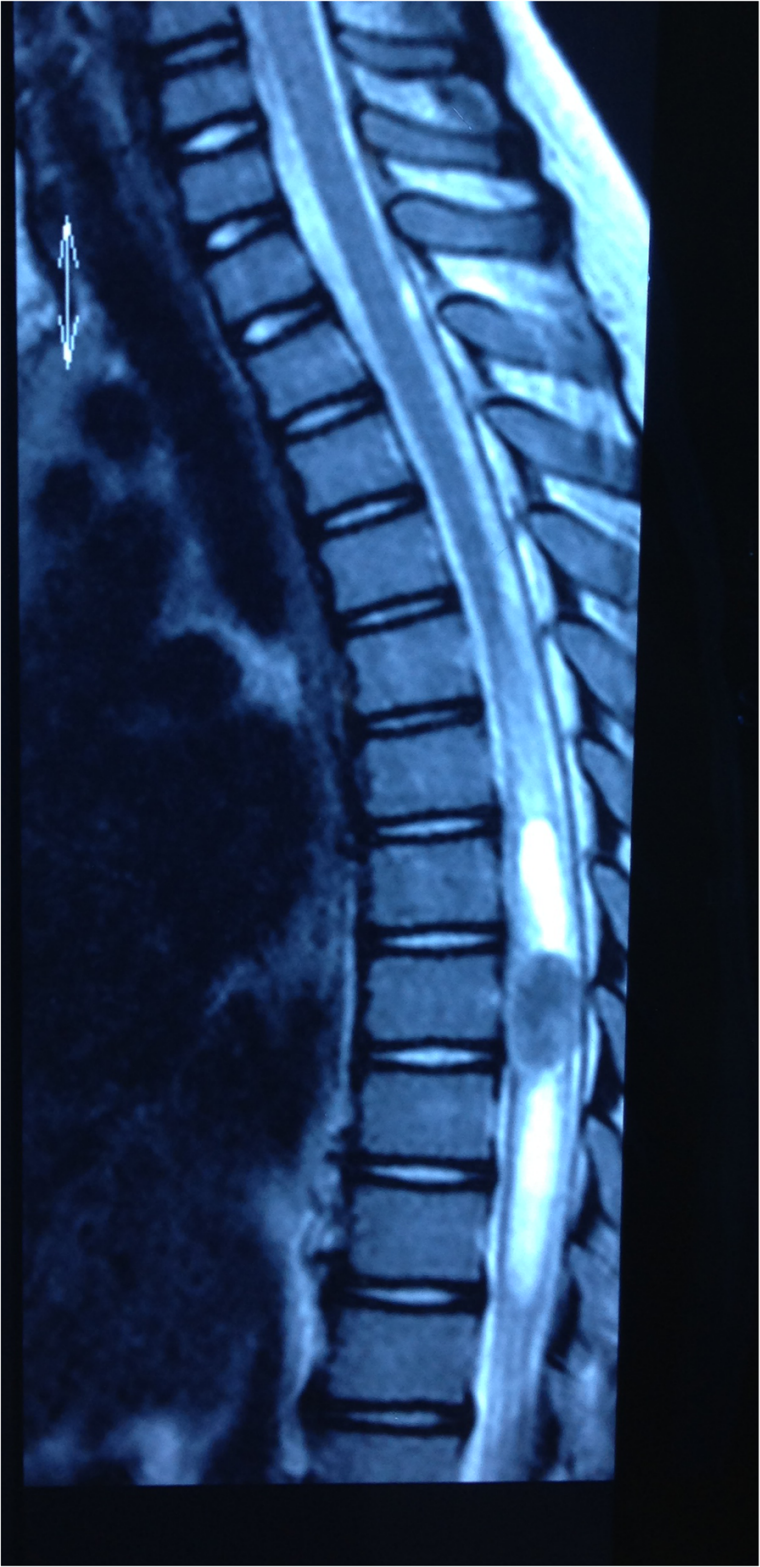
Magnetic resonance imaging (MRI) sagital T2WI showed an well-demarcated intramedullary lesion associated with syringomyelia

**Fig. 2:**
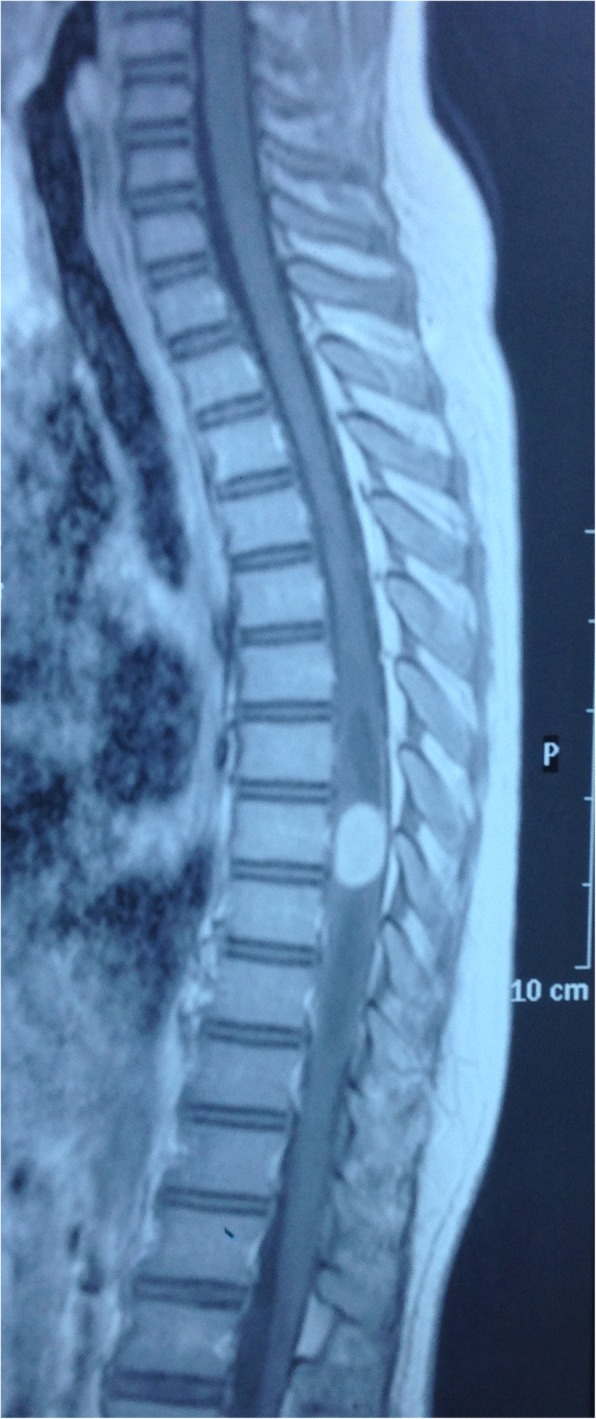
Magnetic resonance imaging (MRI) sagittal T1 with contrast showed an well-demarcated intramedullary lesion associated with syringomyelia

**Fig. 3:**
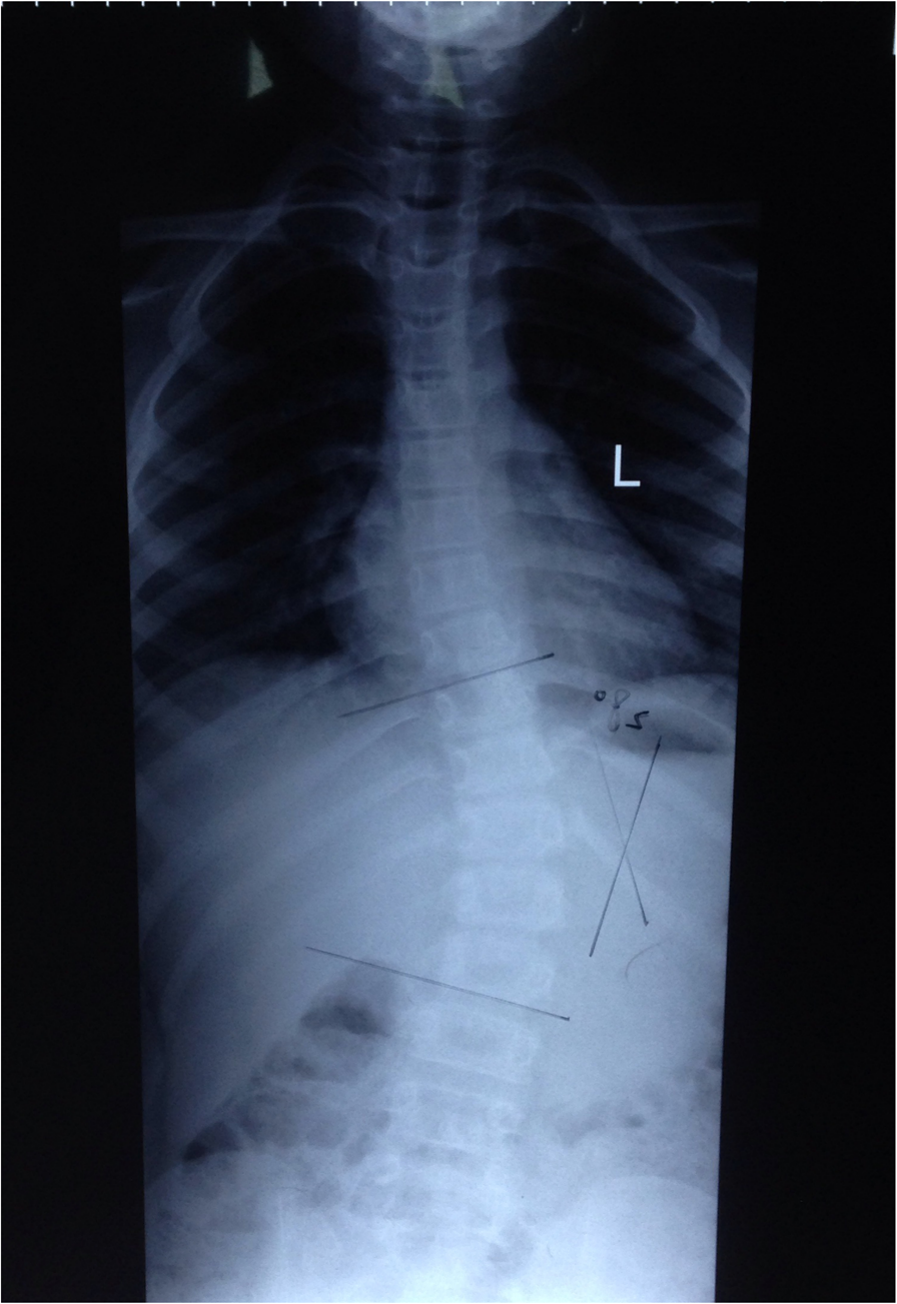
A preoperative scoliotic deformity was also observed

**Fig. 4:**
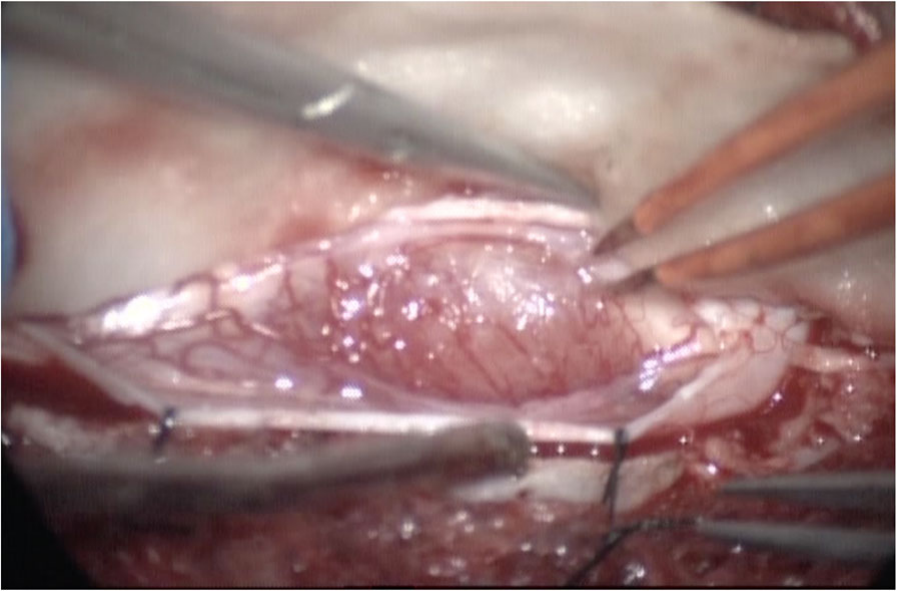
A well-demarcated, soft, greyish-red tumor was observed during operation

**Fig. 5:**
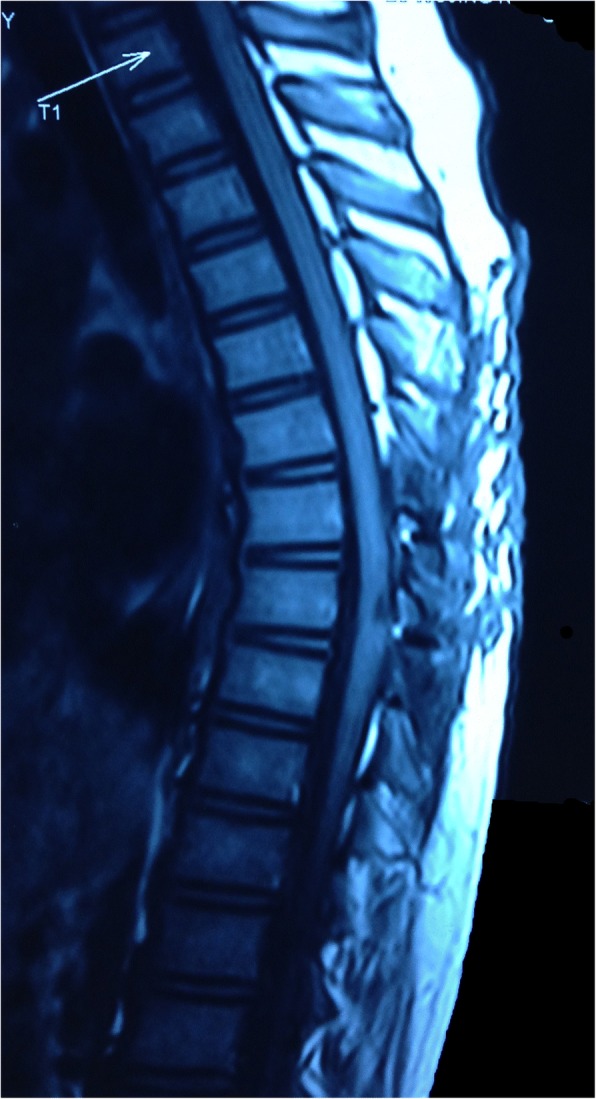
MRI revealed a mild spinal kyphosis 5 months after surgery

**Fig. 6:**
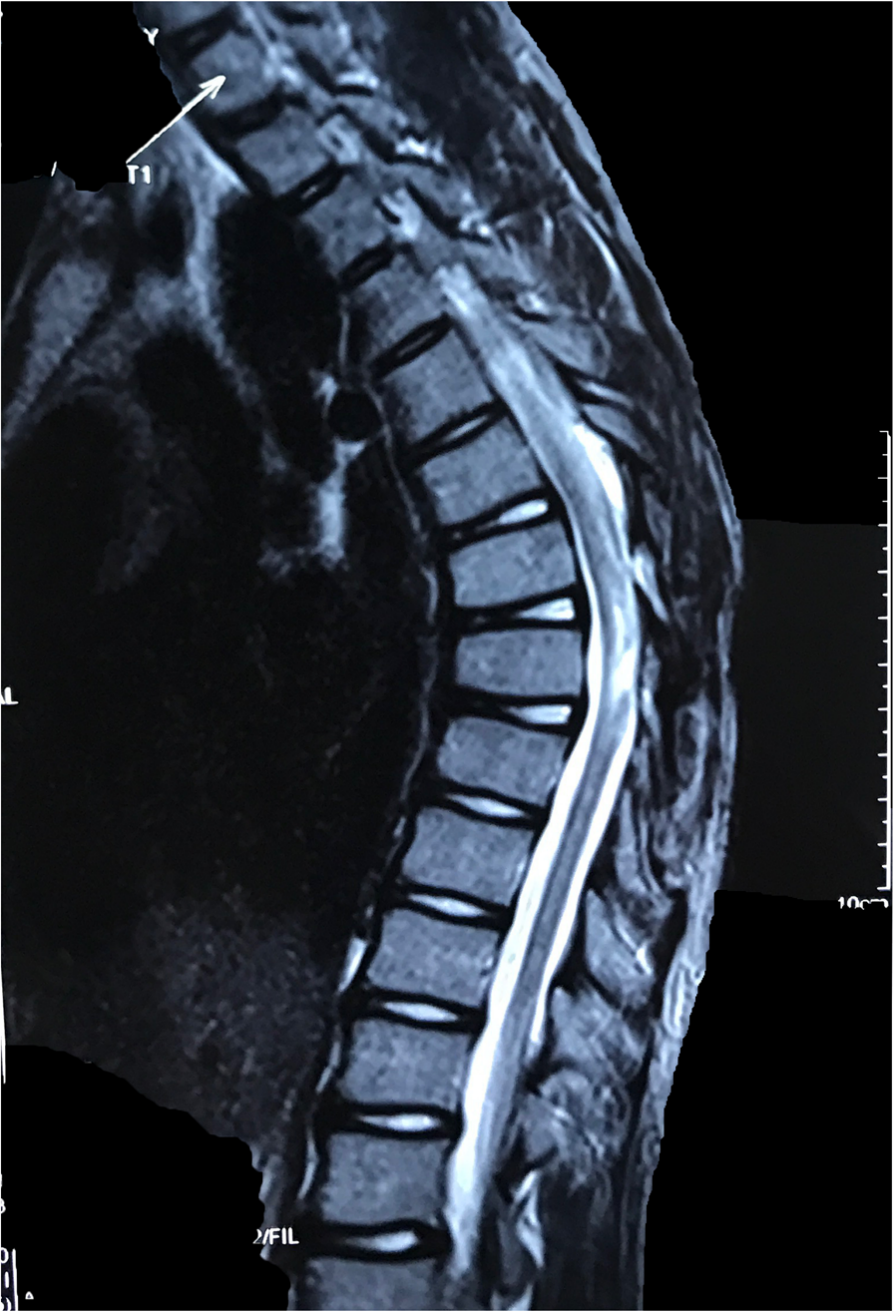
Postoperative MRI did not reveal the presence of a residual tumor and a progressive spinal kyphosis was observed 3 years after surgery

A 9-year-old Han Chinese boy presented with both lower extremity weakness of 6-month duration. Neurophysical examination revealed weakness of the lower extremities (power grade I*V*/V) and decreased sensation below the T10 dermatome with bilateral knee tendon hyperreflexia and Babinski sign positive. Magnetic resonance imaging (MRI) showed an well-demarcated intramedullary lesion located at the level of T8 vertebra with isointensity on T2WI (Fig. 1) and hypointensity on T1WI, which was homogeneous enhanced after gadolinium injection (Fig. 2). There was associated syringomyelia extending from T7 down to the level of T10. A right thoracolumbar scoliosis with a Cobb angle of 28° was also observed (Fig. 3). The patient underwent a T7–8 laminectomy. Opening the dura mater revealed a well-demarcated, soft, greyish-red tumor (Fig. 4). The lesion was totally resected with the help of microsurgical techniques. T7–8 laminoplasty was performed to keep the integrity of spinal structural. Histopathological findings were consistent with fibrillary schwannoma. Postoperative MRI did not reveal the presence of a residual tumor with syringomyelia reduced. By 2 weeks after treatment, the patient had experienced nearly complete recovery. Management with external bracing was performed on this patient for 3 months after surgery to prevent spinal deformity. However, a thoracic kyphotic deformity with a Cobb angle of 30° occurred 5 months after surgery (Fig. 5), and a progressive postoperative spinal kyphosis with a Cobb angle of 60° was observed 3 years after surgery (Fig. 6). Continued conservative management with observation was performed as there is no association with functional decline and impairment in health-related quality-of-life measures.

## Discussion and conclusions

Intramedullary schwannomas are very rare, and only about 60 cases have been reported without neurofibromatosis and generally present in the fourth decade of life [1, 4, 5]. Intramedullary schwannomas is known to occur more in males than in females, and were more commonly seen in the cervical cord [15, 16]. The cause of intramedullary schwannomas is still unknown. There are several hypotheses on the origin of intramedullary schwannomas [17–21]. To date, only eight pediatric cases of intramedullary schwannoma without neurofibromatosis have been reported in the PubMed published English literature [6–13], not including an extensive thoracolumbar congenital intramedullary schwannoma [22]. In the present case, intramedullary schwannoma occurred at the age of nine. By reviewing nine pediatric cases including the present one, age ranged from 8 to 15 years, with a median age of 10 years. The predominance of male (male: female = 7:2) is consistent with the previous literature. The involved vertebral levels of pediatric intramedullary schwannomas are cervical (4/9,44.4%), thoracic (4/9,44.4%) and cervicothoracic (1/9,11.1%) respectively.

MRI is the modality of choice for diagnosing spinal cord tumors. Many imaging studies have been conducted to characterize intramedullary schwannoma. Intramedullary schwannomas are usually isointense or hypointense on T1-weighted images and hyperintense on T2-weighted images [1, 5–10, 15]. Hypointense areas on T2-weighted images add evidence to diagnose intramedullary schwannomas [1, 15]. A well-demarcated tumor with these features, and showing obvious enhancement, with absence of syringomyelia, should raise the possibility of an intramedullary schwannoma [15]. Generally, the absence of syringomyelia is one of characteristic MRI finding of intramedullary schwannomas [2–7, 9, 15]. However, the association of syringomyelia were also reported in some cases of intramedullary schwannoma [1, 6, 8, 10]. Yang et al. [1] reported 9 of 20 cases of intramedullary schwannoma had syringomyelia. In our case, there was associated syringomyelia extending from T7 down to the level of T10. By reviewing 9 pediatric cases including the present one, 4 cases were found associated syringomyelia [6, 8, 10]. We speculate that the syringomyelia was caused by tumor which may obstruct the circulation of cerebrospinal fluid in the central canal. Therefore, we believe that the absence of syringomyelia is common but not specific. Associated syringomyelia can be found but uncommon. The differential diagnosis includes all other type of intramedullary lesions, such as astrocytoma, ependymoma, and hemangioblastoma [4, 15]. However, these lesions have other characteristic imaging features. Most astrocytomas commonly have ill-defined tumor margins with mild enhancement even no enhancement; Intramedullary ependymomas are usually located in the central part of the spinal cord and often associated with syringomyelia; Flow void, hemorrhage, and a typically bright enhancing pattern is evidence in favor of a hemangioblastoma. In addition, these lesions are usually associated with peritumoral edema and tumor cysts. Although Yang et al. [1] have reported that peritumoral edema is uncommon, Gao et al. [15] found most intramedullary schwannomas had different degrees of peritumoral edema. On the other hand, tumor cyst are not uncommon in intramedullary schwannomas. Yang et al. [1] found 55% (11/20) of intramedullary schwannomas with associated cyst formation. Therefore, there is no uniformity on imaging features of intramedullary schwannomas, it may still be difficult to identify based only on MRI.

Total surgical resection should be attempted because of the benign nature of schwannomas [1, 4]. However, total resection may be difficult in some cases [6, 8]. Yang et al. [1] reported 20 cases of intramedullary schwannoma, among of which 16 cases achieved gross total resection (GTR) and 4 cases subtotal resection (STR), and thought a good clinical outcome after GTR or STR can be expectd. Lee et al. [4] reported a 80% (8/10) GTR rate of intramedullary schwannomas. By reviewing reported 8 pediatric intramedullary schwannomas, the GTR rate is 50% (4/8) [7, 9–11]. In our case, the tumor achieved GTR as there is a well-demarcated dissection plane to the surrounding neural tissue. Yang et al. [1] observed syrinx reduction in 77.8% (7/9) of intramedullary schwannoma patients with syringomyelia after the surgery and none with syrinx extension. However, Kim et al. [8] reported a case of pediatric intramedullary schwannoma associated with syringomyelia, and described an extension of syringomyelia after the tumor subtotally resected. In our case, syrinx reduction was observed after GTR of the tumor. Therefore, in view of the syringomyelia secondary to the intramedullary schwannoma, we don’t need to drain the syrinx additionally, because it may collapse after tumor removal. The long-term outcomes of intramedullary schwannomas remains unclear as it’s rarity. Yang et al. [1] reported 20 operated cases and concluded that a good prognosis can be achieved after GTR or STR. By reviewing reported pediatric cases, only one case experienced aggravation 9 months after subtotal resection of the tumor because of the extension of the syrinx [8]. In the present case there was no recurrence or clinical deterioration after 3 years follow-up. However, a progressive postoperative spinal kyphosis was observed during these 3 years of follow-up. Currently, there are some studies on postoperative spinal deformity after resection of intramedullary spinal cord tumors (IMSCTs). Pediatric patients with preoperative kyphoscoliosis or tumor-associated syrinx or surgery spanning more than 4 levels should be monitored for development of spinal deformity [23, 24]. In our case, progressive postoperative spinal kyphosis may be related to these risk factors.

Although extremely rare and uncommonly associated with syringomyelia, schwannomas need to be considered in the preoperative diagnosis of solitary intramedullary tumors in children as total resection can be achieved improving surgical outcome; Pediatric patients should be monitored closely for the development of spinal deformity following resection of intramedullary schwannoma, particularly possessing preoperative scoliotic deformity and/or tumor-associated syringomyelia.

## Abbreviations

GTR: Gross total resection
IMSCTs: Intramedullary spinal cord tumors
MRI: Magnetic resonance imaging
STR: Subtotal resection
T2WI: T2-weighted images
TIWI: T1-weighted images
